# Association between D-dimer level and chest CT severity score in patients with SARS-COV-2 pneumonia

**DOI:** 10.1038/s41598-021-91150-1

**Published:** 2021-06-02

**Authors:** Lan Wang, Ling Yang, Lang Bai, Zhixin Huang, Yong Peng

**Affiliations:** 1 Department of Respiratory and Critical Care Medicine, West China Hospital, Sichuan University, #37 Guoxue Alley, Chengdu, 610041 People’s Republic of China; 2 Department of Cardiology, West China Hospital, Sichuan University, #37 Guoxue Alley, Chengdu, 610041 People’s Republic of China; 3 Center of Infectious Diseases, West China Hospital, Sichuan University, #37 Guoxue Alley, Chengdu, 610041 People’s Republic of China; 4 Department of Obstetrics and Gynecology, Renmin Hospital of Wuhan University, #238 Jiefang Road, Wuchang District, Wuhan, 436000 People’s Republic of China

**Keywords:** Diseases, Medical research

## Abstract

The elevated level of D-dimer and its relationship with poor outcomes in SARS-COV-2 pneumonia patients have been demonstrated. In addition to a hypercoagulable state, D-dimer is also a biomarker of inflammation. We investigated the relationship between D-dimer level and chest computed tomography (CT) severity score, which could reflect the severity of inflammation in SARS-COV-2 pneumonia patients. We retrospectively enrolled 86 consecutive SARS-COV-2 pneumonia patients. CT severity scores were computed to quantify the overall lung involvement. The D-dimer level among CT score tertiles and the association of the D-dimer level with CT score were analyzed. Our results showed that the median D-dimer level was 0.70 mg/L (IQR 0.35–1.76). 42 patients (48.8%) had D-dimer levels above the median level. The D-dimer levels were significantly different across CT score tertiles (0.37 mg/l [IQR 0.31–0.87], 0.66 mg/l [IQR 0.39–1.43], 1.83 mg/l [IQR 0.85–4.41], P < 0.001). The natural logarithm of the D-dimer level was significantly associated with the CT score (r_s_ = 0.586, P < 0.001). In conclusion, the D-dimer level may be associated with the severity of inflammation of SARS-COV-2 pneumonia prior to coagulopathy/thrombosis. This could be an additional explanation for the mechanism of the relationship between elevated D-dimer level and higher mortality.

## Introduction

Since the outbreak of coronavirus disease 2019 (COVID-19) worldwide, studies from different countries have consistently found elevated levels of D-dimer in patients with SARS-COV-2 pneumonia^1,2^. Furthermore, several studies also demonstrated that a higher level of D-dimer was associated with in-hospital mortality^3,4^. The most suggested mechanism was that the hypercoagulable state, which could be reflected by an elevated D-dimer level, might lead to thrombotic events, resulting in poor outcomes. However, the coagulopathy was thought to result from local and systemic inflammation caused by the coronavirus. Also, D-dimer is known as a biomarker of inflammation^5^. Therefore, we proposed the hypothesis that the D-dimer level may be associated with the severity of inflammation rather than directly related to the hypercoagulable state in patients with SARS-COV-2 pneumonia. Chest computed tomography (CT) involvement extent is the most visual parameter, which could reflect the severity of inflammation^6^. In this study, we investigated the relationship between D-dimer level and CT severity score in patients with SARS-COV-2 pneumonia.

## Results

### Comparison of clinical characteristics between patients dichotomized by the 50th percentile of D-dimer level

Of the 98 patients confirmed SARS-COV-2 pneumonia, 86 (87.8%) patients was finally included in this study. Their median age was 61 years (IQR 47–69), and 50 (58.1%) of them were female. The median D-dimer level was 0.70 mg/L (IQR 0.35–1.76). 42 patients (48.8%) had D-dimer level above the median level (0.7 mg/L). Patients with D-dimer level above 0.7 mg/L had older age (67 yrs [IQR 60.2–73.8] vs. 51 yrs [IQR 42.5–63.0]; P < 0.001), higher level of C-reactive protein (35.8 mg/L [IQR 5.0–95.6] vs. 5.6 mg/L [IQR 5.0–29.8]; P = 0.017) and NT-proBNP (200.4 pg/ml [IQR 91.6–380.8] vs. 32.6 pg/ml [IQR 18.0–91.9]; P < 0.001) than the ones with D-dimer level ≤ 0.7 mg/L. Furthermore, there were more patients received oxygen therapy in the group with D-dimer level > 0.7 mg/L than ≤ 0.7 mg/L (73.8% vs. 45.5%, P = 0.009). Patients with D-dimer level > 0.7 mg/L had lower oxygenation index compared with patients with D-dimer level ≤ 0.7 mg/L (273.2 [IQR 202.4–332.4] vs. 337.3 [IQR 292.4–385.4], P < 0.047). There were more patients had oxygenation index < 300 in in the group with D-dimer level > 0.7 mg/L than ≤ 0.7 mg/L (57.6% vs. 25%, P = 0.043) (Table 1).

**Table 1:** Comparison of clinical characteristics between patients with D-dimer ≤ 0.7 mg/L and > 0.7 mg/L.

Characteristic	All patients (N = 86)	D-dimer ≤ 0.70 mg/L (N = 44)	D-dimer > 0.70 mg/L (N = 42)	*P* value
Age, yrs	61.0 (47.0–68.8)	51.0 (42.5–63.0)	67.0 (60.2–73.8)	< 0.001
Female, n (%)	50 (58.1%)	24 (54.5%)	26 (61.9%)	0.636
**Coexisting illnesses**				
Hypertension, n (%)	18 (20.9%)	4 (9.1%)	14 (33.3%)	0.013
Diabetes, n (%)	4 (4.7%)	2 (4.5%)	2 (4.8%)	1.000
IHD, n (%)	1 (1.2%)	1 (2.3%)	0 (0.0%)	1.000
COPD, n (%)	4 (4.7%)	2 (4.5%)	2 (4.8%)	1.000
Cancer	3 (3.5%)	1 (2.3%)	2 (4.8%)	0.612
**Vital signs**				
Temperature, °C	36.5 (36.4–36.7)	36.5 (36.4–36.6)	36.5 (36.4–36.7)	0.409
Respiratory rate, per min	20.0 (18.0–21.0)	20.0 (18.0–21.0)	20.0 (18.0–21.0)	0.308
Heart rates, beats per min	86.0 (78.0–96.0)	90.0 (78.0–98.0)	82.0 (78.0–94.0)	0.295
SBP, mmHg	128.0 (120.0–136.0)	122.0 (113.0–132.0)	131.0 (124.0–142.2)	0.003
DBP, mmHg	78.0 (73.0–83.0)	76.0 (72.0–80.0)	80.0 (75.0–87.0)	0.058
**Laboratory findings**				
White cell count, per mm^3^	5.300 (4.135–6.537)	5.075 (3.748–5.938)	6.075 (4.598–7.282)	0.006
Hemoglobin, g/liter	124.0 (114.0–135.5)	125.0 (115.0–138.0)	120.0 (110.2–131.8)	0.105
Hematocrit	0.36 (0.32–0.39)	0.37 (0.35–0.40)	0.34 (0.32–0.38)	0.030
Platelet count, per mm^3^	234,000 (172,500–282,750)	234,000 (169,500–272,250)	234,500 (184,750–289,750)	0.473
D-dimer	0.70 (0.35–1.76)	0.36 (0.26–0.46)	1.78 (1.17–4.40)	< 0.001
CD4 + cell	452 (263–586)	449 (350–568)	488 (229–589)	0.718
CD8 + cell	244 (154–380)	290 (197–434)	209 (93–340)	0.016
Creatinine, μmol/liter	59.5 (48.0–70.8)	60.5 (49.5–72.0)	58.5 (47.0–69.0)	0.595
ALT, U/liter	22.0 (15.0–37.8)	22.5 (16.5–39.2)	21.0 (15.0–30.2)	0.675
AST, U/liter	24.0 (18.0–34.0)	24.5 (17.8–31.2)	23.5 (18.2–37.8)	0.653
Albumin, g/liter	37.7 (34.741.0)	39.8 (37.8–42.0)	35.4 (32.2–37.5)	< 0.001
Sodium, mEq/L	142.0 (138.0–145.0)	142.0 (140.0–144.2)	142.0 (137.2–145.0)	0.742
Procalcitonin, ng/mL	0.04 (0.03–0.07)	0.04 (0.03–0.06)	0.05 (0.03–0.09)	0.277
CRP, mg/liter	11.5 (5.0–46.6)	5.6 (5.0–29.8)	35.8 (5.0–95.6)	0.017
CK-MB, U/liter	1.0 (0.6–1.4)	0.9 (0.6–1.2)	1.1 (0.9–1.8)	0.005
hs-cTnI > 99th percentile URL	8 (9.4%)	3 (7.0%)	5 (11.9%)	0.483
NT-proBNP, pg/ml	88.0 (30.9–248.9)	32.6 (18.0–91.9)	200.4 (91.6–380.8)	< 0.001
NT-proBNP > 300 pg/ml	20 (23.3%)	4 (9.1%)	16 (38.1%)	0.003
Oxygen therapy, n (%)	51 (59.3%)	20 (45.5%)	31 (73.8%)	0.009
High-flow nasal cannula	2 (2.3%)	1 (2.3%)	1 (2.4)	1.000
Non-invasive respiratory support	3 (3.5%)	0 (0%)	3 (7.1%)	0.112
Invasive respiratory support	1 (1.2%)	0 (0%)	1 (2.4%)	0.488
Saturaion, %^#^	97 .0 (96.0–99.0)	97.5 (96.0–99.0)	97.0 (96.0–99.0)	0.318
Oxygenation index*	308.1 (224.2–376.0)	337.3 (292.4–385.4)	273.2 (202.4–332.4)	0.047
Oxygenation index < 300*	24 (45.3%)	5 (25.0%)	19 (57.6%)	0.043

Data are expressed as median (interquartile range) or counts and percentage, as appropriate.

*IHD* Ischemic heart disease, *COPD* Chronic obstructive pulmonary disease, *SBP* Systolic blood pressure, *DBP* Diastolic blood pressure, *ALT* Alanine aminotransferase, *AST* Aspartate aminotransferase, *CRP* C-reactive protein, *CK-MB* Creative kinase MB, *hs-cTnI* high-sensitivity cardiac troponin I, *URL* upper reference limit, *NT-proBNP* N-terminal pro-B-type natriuretic peptide.

^#^There were 28 patients in the group of D-dimer ≤ 0.70 mg/L and 33 patients in D-dimer > 0.70 mg/L had the data of Saturation.

*There were 19 patients in the group of D-dimer ≤ 0.70 mg/L and 31 patients in D-dimer > 0.70 mg/L had the data of oxygenation.

### Comparison of radiographic findings between patients with D-dimer ≤ 0.7 mg/L and > 0.7 mg/L

**Figure 1:**
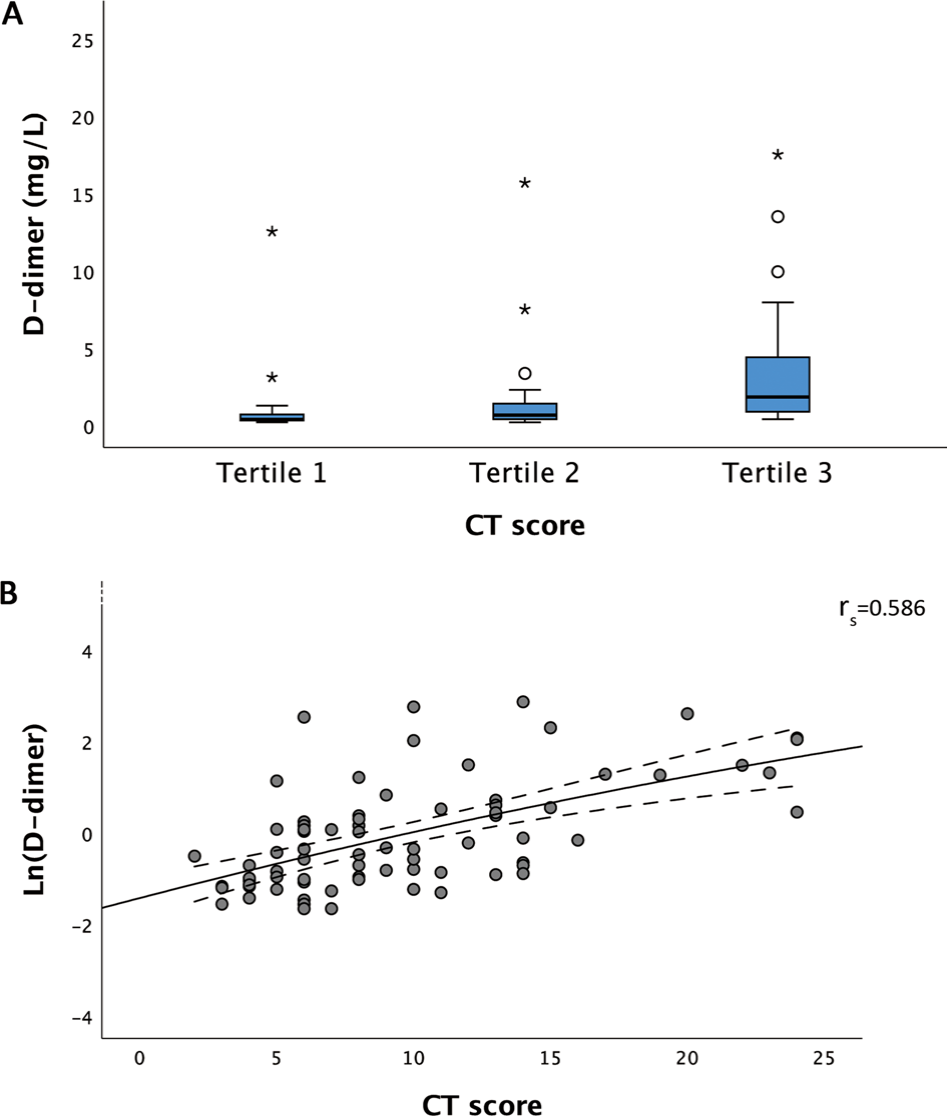
(**A**) The boxplot showing a significant difference in D-dimer levels across CT score tertiles (P < 0.001). (**B**) The scatterplot was showing that the natural logarithm of the D-dimer level was significantly associated with CT score (r_s_ = 0.586, P < 0.001).

A total of 76 patients had a high-resolution chest CT scan during the study period. The median CT score was 8.0 (IQR 6.0–13.0). Patients with D-dimer level > 0.7 mg/L had significantly higher CT score (12.0 [IQR 8.0–15.0] vs. 6.0 [IQR 4.0–9.5], P < 0.001) and higher incidence of reticulation and/or traction bronchiectasis on chest CT images (83.3% vs. 46.2%, P = 0.002) than patients with D-dimer level ≤ 0.7 mg/L (Table 2). The D-dimer levels were significantly different across CT score tertiles (0.37 mg/l [IQR 0.31–0.87], 0.66 mg/l [IQR 0.39–1.43], 1.83 mg/l [IQR 0.85–4.41], for tertile 1–3, respectively; P < 0.001) (Fig. 1A). The natural logarithm of the D-dimer level was significantly associated with the CT score (r_S_ = 0.586, P < 0.001) (Fig. 1B).

**Table 2:** Comparison of radiographic findings between patients with D-dimer ≤ 0.7 mg/L and > 0.7 mg/L.

Radiographic finding	All	D-dimer ≤ 0.70 mg/L	D-dimer > 0.70 mg/L	*P* value
	(n = 76)	(n = 39)	(n = 37)	
CT score	8.0 (6.0–13.0)	6.0 (4.0–9.5)	12.0 (8.0–15.0)	< 0.001
More than two lobes involvement	75 (98.7%)	38 (97.4%)	37 (100.0%)	1.000
Pleural effusion	2 (2.6%)	0 (0.0%)	2 (5.4%)	0.234
Ground-glass opacity	74 (98.7%)	38 (97.4%)	36 (100.0%)	1.000
Consolidation	55 (73.3%)	29 (74.4%)	26 (72.2%)	1.000
Reticulation and/or traction bronchiectasis	48 (64.0%)	18 (46.2%)	30 (83.3%)	0.002

Data are expressed as counts and percentage. The Pearson Chi-square or Fisher's Exact Test was used to test the difference between D-dimer groups, as appropriate.

*HRCT* high resolution computed tomography.

### Comparison of clinical events between patients with D-dimer ≤ 0.7 mg/L and > 0.7 mg/L

Patients with D-dimer level > 0.7 mg/L had a higher rate of mechanical ventilation (14.3% vs. 0.0%, P = 0.011) and higher incidence of composite endpoint (14.3% vs. 0.0%, P = 0.011) than the ones with D-dimer level ≤ 0.7 mg/L. Four patients had pulmonary thromboembolism (PE) in this study, whose D-dimer levels were all above 0.7 mg/L (Table 3). There was one patient died during the study period who had segmental PE.

**Table 3:** Comparison of clinical events between patients with D-dimer ≤ 0.7 mg/L and > 0.7 mg/L.

Clinical events	All	D-dimer ≤ 0.70 mg/L	D-dimer > 0.70 mg/L	*P* value
	(n = 86)	(n = 44)	(n = 42)	
Thrombotic events	4 (4.7%)	0 (0.0%)	4 (9.5%)	0.053
Mechanical ventilation requirement	6 (7.0%)	0 (0.0%)	6 (14.3%)	0.011
ECMO requirement	2 (2.3%)	0 (0.0%)	2 (4.8%)	0.236
All-cause Death	1 (1.2%)	0 (0.0%)	1 (2.4%)	0.488
Composite endpoint	6 (7.0%)	0 (0.0%)	6 (14.3%)	0.011

Data are expressed as counts and percentages. The Pearson Chi-Square or Fisher's Exact Test was used to test the difference between D-dimer groups, as appropriate.

*ECMO* extracorporeal membrane oxygenation; the composite endpoint was the composite of mechanical ventilation requirement, ECMO requirement, or all-cause death.

## Discussion

In our study, the median D-dimer level of total patients was 0.7 mg/L. This is close to the level previously reported by Zhou et al. (0.8 mg/L)^2^ in Wuhan but lower than the one by Cummings et al. in New York (1.6 ug/ml)^4^, whose study population were all critical cases. There were 48.8% of patients in our study had D-dimer levels > 0.7 mg/L. Their chest CT severity scores were higher than those with D-dimer level ≤ 0.7 mg/L, and more patients (83.3%) had reticulation and/or traction bronchiectasis in the Chest CT imaging. Furthermore, we also found that the D-dimer level was well correlated with the chest CT score. Since a recent study has proved the chest CT score could be an imaging tool for assessing the severity of SARS-COV-2 pneumonia^7,8^, our findings would lead us to conclude that D-dimer level could be a biomarker for severe SARS-COV-2 pneumonia by paralleling with the inflammation involvement extent in lungs which was reflected by chest CT score. The more frequent presence of reticulation and/or traction bronchiectasis in these patients was also evidence of more extensive and severe inflammation response and lung injury. Considering the limited sample size of our study, we didn’t analyze the relationship between the D-dimer level and CT score adjusted for age. Further researches with a larger size are needed to investigate their relationship adjusted for age.

The relationship between D-dimer level and coagulopathy/thrombosis in COVID-19 patients has been widely confirmed. However, whether the time points of D-dimer elevation and coagulopathy/thrombosis present were consistent has not been fully addressed. Based on our findings in which the D-dimer level's time point was matched to the time of CT scan, we have reasons to speculate that the D-dimer level may be associated with the severity of inflammation prior to coagulopathy/thrombosis. Uncontrolled inflammation response itself could result in severe lung injury and sequentially aggravate coagulopathy/thrombosis and then lead to poor outcomes, even death. This could be an additional explanation for the mechanism of the relationship between elevated D-dimer and higher mortality which previous studies have demonstrated. Further researches are needed to investigate the relationship between the dynamics of D-dimer level and the severity of inflammation and coagulopathy.

## Methods

For this retrospective, single-center study, we enrolled consecutive patients from February 9 to March 4, 2020, in a COVID-19 ward of Renmin Hospital of Wuhan University (East Branch) in Wuhan, which is a government-assigned center for COVID-19 treatment. The diagnosis was confirmed by microbiological and radiographic findings following the World Health Organization (WHO) interim guidance^9^ and the Fifth Revised Trial Version of the Novel Coronavirus Pneumonia Diagnosis and Treatment Guidance in China^10^. The observed time of this study ended on April 7. Only the patients who had D-dimer results during the study period were included. The study was approved by the Clinical Rearech Ethics Committee of West China Hospital (2020-226), and the requirement for written informed consent was waived by the Ethics Committee.

Demographic data, vital signs, laboratory test reports on admission, and outcomes were obtained from the patients' electronic medical records and scrutinized by investigators. Attending physicians in respiratory medicine reviewed the first high-resolution chest CT images of the patients for analyzing the radiographic patterns such as ground-glass opacities (GGOs), consolidation, reticulation, traction bronchiectasis, and pleural effusion, and for computing CT severity score to quantify overall lung involvement. Each lung was divided into three lung zones. Each lung zone (total of six lung zones) was assigned a score that was based on the following: score 0, 0% involvement; 1, 0–25%; 2, 25–50%; 3, 50–75%; and 4, > 75%. Summation of scores provided overall lung involvement (maximal CT score for both lungs was 24). D-dimer levels at the time point most nearby the date of CT were obtained in patients with high-resolution chest CT. D-dimer testing was performed using the Siemens Innovance D-Dimer Assay. According to the manufacturer's instructions, a value of 0.55 mg/L fibrinogen equivalent units (FEU) was selected as the cut-off value of the normal range with a sensitivity of ≥ 98%.

Data were expressed as the median and interquartile range (IQR [25th to 75th percentiles]) for continuous variables due to the skewed distribution (tested by the Shapiro–Wilk normality test) and as count and percentage for categorical variables. T-test or Mann–Whitney U test was used to analyze the differences between two groups dichotomized by the 50th percentile of D-dimer level for continuous variables. χ^2^ test was used for categorical variables. The difference of D-dimer levels among CT score tertiles was tested using the Kruskal–Wallis test. Spearman's correlation coefficient was used to measure the association of the natural logarithm of the D-dimer level with CT score. All the statistical analyses were performed with the use of IBM SPSS Statistics software (version 26.0, https://www.ibm.com/support/pages/downloading-ibm-spss-statistics-26).

All the methods above were carried out in accordance with relevant guidelines and regulations.
